# Orbivirus Screening from Imported Captive Oryx in the United Arab Emirates Stresses the Importance of Pre-Import and Transit Measures

**DOI:** 10.3390/pathogens11060697

**Published:** 2022-06-17

**Authors:** Ludovic Martinelle, Andy Haegeman, Louis Lignereux, Anne-Lise Chaber, Fabiana Dal Pozzo, Ilse De Leeuw, Kris De Clercq, Claude Saegerman

**Affiliations:** 1 CARE-FEPEX Experimental Station, Fundamental and Applied Research for Animals & Health (FARAH) Center, Faculty of Veterinary Medicine, University of Liege, 4000 Liege, Belgium; l_louis77@hotmail.com (L.L.); fdalpozzo@uliege.be (F.D.P.); claude.saegerman@uliege.be (C.S.); 2 Sciensano, Infectious Diseases in Animals, Exotic and Particular Diseases, 1050 Brussels, Belgium; andy.haegeman@sciensano.be (A.H.); ilse.deleeuw@sciensano.be (I.D.L.); kris.declercq@sciensano.be (K.D.C.); 3 School of Animal and Veterinary Sciences, University of Adelaide, Adelaide, SA 5005, Australia; anne-lise.chaber@adelaide.edu.au

**Keywords:** bluetongue, vector-borne disease, orbivirus, arboviruses, oryx, biosecurity

## Abstract

From 1975 to 2021, the United Arab Emirates (UAE) imported more than 1300 live Arabian oryxes (AOs) and scimitar-horned oryxes (SHOs) for conservation programs. The objective of this study was to estimate the prevalence of orbiviruses Bluetongue virus (BTV) and epizootic hemorrhagic disease virus (EHDV) in AOs and SHOs from captive herds in the UAE. Between October 2014 and April 2015, 16 AOs and 13 SHOs originating from Texas (USA) and 195 out of about 4000 SHOs from two locations in the UAE were blood sampled to be tested by indirect enzyme-linked immunosorbent assay (ELISA) and real-time reverse transcriptase polymerase chain reaction (RT-qPCR) assays. Eight imported AOs (50% CI [24.7–75.4%]) and eight imported SHOs (61.5% CI [31.6–86.1%]) were found BTV seropositive, in contrast with three out of 195 SHOs (1.5% CI [0.3–4.4%]) from the Emirates. BTV-2 genome was detected in 6/16 of the Arabian Oryx, and amongst those, one out of six was seronegative. None of the tested samples was found positive for EHDV. Our results illustrate the wide local variation regarding BTV seroprevalence in domestic and wild ruminants in the Arabian Peninsula. These results stress the need for pre-import risk assessment when considering translocation of wild ruminant species susceptible to orbiviruses not only in the country of destination but also where transit happens.

## 1. Introduction

Bluetongue virus (BTV) and epizootic hemorrhagic disease virus (EHDV) are vector-borne RNA viruses belonging to the genus *Orbivirus*, family *Reoviridae* that are the causative agents of bluetongue disease (BT) and epizootic hemorrhagic disease (EHD), respectively [1]. Both viruses are transmitted to host species by the bite of hematophagous midges of the genus *Culicoides* (Diptera: Ceratopogonidae) [2]. All ruminants are susceptible to infection with BTV; clinical disease is most often observed in sheep, whereas cattle (and goats) are considered reservoir species. In wildlife, a serious disease develops in white-tailed deer (*Odocoileus virginianus*) and pronghorn antelope (*Antilocapra americana)* [3,4,5]. EHDV severe infections, by contrast, are mostly limited to white-tailed deer despite some sporadic serious cases in other ruminant species. In susceptible livestock and other wildlife, the disease is considered generally subclinical [6]. When present, clinical manifestations of both diseases are quite similar, from a mild non-specific clinical picture including hyperthermia, weakness, depression, and anorexia to a fulminant hemorrhagic disease syndrome [6,7], possibly leading to dramatic economic losses [8]. In addition to the affected host species, virulence depends on the virus strains, serotypes, and geographical origin [9]. So far, up to 36 BTV serotypes have been described, including historical serotypes (BTV-1 to BTV-24) and the more recent non-virulent BTV-25 to BTV-36 [10,11]. There are currently at least seven EHDV serotypes [12,13].

The Arabian oryx (AO, *Oryx leucoryx*) and the scimitar-horned oryx (SHO, *Oryx dammah*) are two of six surviving species within the subfamily Hippotraginae [14]. These antelopes were endemic to the Arabian Peninsula and North Africa, respectively. Massive poaching and destruction of the habitat of the AOs and SHOs led to the extinction of these species in the wild in the 1970s for the AO and in the late 1980s or early 1990s for the SHO [15,16]. Reintroduction and conservation programs of the AO and SHO in the Middle East significantly rely on the use of captive-bred animals to be released into their former ranges [14]. The import of semi-wild animals in the United Arab Emirates (UAE) from game ranches in the United States of America (USA) is not uncommon, and Texas is one of the states providing AOs and SHOs to the UAE for conservation purposes. Based on the CITES database (https://trade.cites.org/en/cites_trade/#, accessed on 14 October 2021) and prioritizing the numbers reported by the exporters, there were 5814 SHOs and AOs live-traded globally between 1975 and 2021. The UAE was the main importer (1325 oryx), preceding Qatar (1066 oryx), Oman (635 oryx), Saudi Arabia (621 oryx), and Jordan (226 oryx). The UAE was also the largest exporter (2758 oryx). The second largest exporter was the USA, and out of the 1469 oryx exported by this country, 1000 were addressed to the UAE. Oryx species, therefore, represent the majority of the 1898 CITES-listed live Bovidae that have been shipped from the USA to the UAE since 1975. Over the last ten years (2011–2021), 195 oryx (76 SHOs and 119 AOs) were officially shipped on five different occasions from the USA to the UAE.

In the aftermath of the unexpected 2006–2012 BTV-8 and 2007–2010 BTV-1 epizootics, by 2015, Western Europe was facing the re-emergence of BTV-8 in France [17]. BTV-8 is believed to have caused greater economic damage than any previous single serotype BTV outbreak [8]. Indeed, it showed an increased severity toward cattle [18] and displayed a noticeable ability to be vertically transmitted [19]. In addition, BTV-1, 2, 4, 9, and 16 have been continuously present in parts of the Mediterranean Basin, including several EU member states, since at least 1998 [20]. These BTV strains originated from the Near and Middle East, where they have been identified since the 1960s and have persisted since [21]. 

BTV-11, 13, and 17 were regularly reported in Texas [22]. BTV-2 has been considered enzootic in the southeastern US since the early 1980s and, more recently, was isolated in California [23,24]. Comparing BTV serotypes possibly found in Europe, southwestern USA, and the Middle East, BTV-2 happens to be potentially present in all three areas. Significant levels of EHDV antibodies were also recorded in wild ruminants both in the Arabian Peninsula [14] and Texas (serotypes 1 and 2 [25]). 

In the current study, we investigated the prevalence of BTV and EHDV in AOs and SHOs imported from Texas to the UAE with a stopover in the European Union in 2013 and 2015 and compared them to the prevalence found in indigenous captive animals. Viruses that were detected were further characterized.

## 2. Results

All results are summarized in Table 1, sorted by species and origin. Test results are expressed as the number of positive animals/tested animals.

### 2.1. Serology

#### 2.1.1. Indigenous Animals

Of the 176 SHOs tested with iELISA at the Abu Dhabi location (site A), three were found to be BTV seropositive (1.7%; 95% CI: 0.3–4.9) and none out of the 19 (0%; 95% CI: 0–17.7) from the Dubai collection (site B). 

#### 2.1.2. Imported Animals

Eight out of the sixteen AOs (50%; 95% CI: 24.7–75.4) and eight out of the thirteen SHOs (61.5%; 95% CI: 31.6–86.1) from the USA were found BTV seropositive.

None of the tested samples could be found positive for EHDV.

### 2.2. Pan-BTV RT-qPCR and Serotype-Specific RT-qPCR

No positive samples were detected in indigenous animals. The BTV genome was detected in 6/16 of the AOs from Texas (38%; 95% CI: 18–61), and amongst those, one out of six was seronegative. Overall, the Cp values were high (33–39). No viral genome could be detected in the SHO samples. 

All six AO samples positive by RT-qPCR were found positive for BTV-2 by serotype-specific RT-qPCR. Among those, two samples were confirmed to be BTV-2 by sequencing, including the seronegative sample. As none of the animals could be found seropositive against EHDV, no further EHDV genome detection was carried out.

## 3. Discussion

The low seroprevalence and viral RNA detection for BTV, as well as the absence of EHDV detection, we observed in the animals from the two locations within the UAE somewhat contrasts the rather elevated seroprevalences reported by Frölich et al. (2005) in AOs (up to 48 and 50% for BTV and EHDV, respectively). These discrepancies might originate from the different natural habitats affecting the local *Culicoides* populations and dynamics. Indeed, Frölich et al. (2005) also reported negative sample collections from specific locations for both viruses. Our results suggest that the UAE’s local natural conditions where the two collections were realized are inadequate for the survival or transmission of orbiviruses. 

On the other hand, 55% of the animals imported from Texas overall were BTV seropositive (respectively, 50% and 61.5% for AO and SHO). In the current study, BTV antibodies were detected using an indirect ELISA kit advised by the manufacturer to be used on milk [26,27]. The preliminary results suggested better correspondence with cELISA on serum when compared to homologous blood samples on dried blood spots tested with cELISA and sELISA [28].

Despite the quite high seroprevalence in both AO and SHO from Texas, the BTV genome was found in 6/16 AOs but 0/13 SHO. BTV RNA is known for being detectable for months following infection in ruminants, up to 213 days in cattle [29], and up to 89 days at the least in sheep [30], although the BTV-25 genome could be detected for 19–25 months in goats [31]. In addition, in red deer, it could be detected for up to 112 days [32], but the length of RNAemia in AO and SHO remains unknown. The positive low-level viral RNA detection in AO suggests a potential outbreak several weeks or months earlier. In the SHO, by contrast, the negative PCR results are likely related to an older infection. 

A BTV-2 PCR-positive sample happened to be seronegative. The iELISA used in the current study detects circulating antibodies targeting VP7. The lack of anti-VP7 antibodies following BTV vaccination or infection was previously reported in cattle and was additionally only poorly correlated to clinical and virological protection [33,34]. A weak positive PCR result and the absence of detectable VP7 antibodies could also be related to a very early stage of infection; however, given the serological and virological status of the other AO, this hypothesis is unconvincing. BTV-2 is not considered the most prevalent serotype in Texas. Neutralizing antibodies to BTV-2 were observed nonetheless in Texas in 1991–1992 [35]. Previous exposures to other BTV serotypes cannot be excluded as BTV-13, BTV-11, and BTV-17 are considered enzootic in Texas [36]. Moreover, BTV-12 and BTV-3 were isolated in Texas in 2008 and 2014, respectively [37]. 

No positive EHDV samples could be found either from USA or UAE. Previous reports showed an EHDV seroprevalence in white-tailed deer widespread throughout Texas, with up to 100% of the tested animals being positive [35]. In 2014, EHDV-2 was successfully isolated in Eld’s deer (*Panolia eldii*) [38]. In Texas, EHDV-2 is the most prevalent serotype, followed by EHDV-1 and the more recent identification of EHDV-6 [39]. In the Middle East, outbreaks of EHDV-6 were reported in 2006, along with EHDV-7 outbreaks in Israel. In 2015, clinical EHDV-6 outbreaks were also reported in Israel [40]. As for BTV, EHDV seroprevalence demonstrated large local variations within a considered country [14].

Since BTV is prevalent in certain areas of the UAE, a transmission occurring between landing and testing cannot be totally ruled out. This case demonstrates that import health permits are not requested by countries where a stopover is carried out, possibly posing a risk of vector-borne disease transmission. Usually on long distance, flight between continents freight is routed to local hubs to be sorted to the final destination (P. Lignereux, personal communication). This implies to take the animals off and reload them in a second plane. Freight transfer only takes a couple of hours with limited contact of the animals with the environment. European Palearctic *Culicoides* species were proven to be competent orbivirus vectors with some species displaying a strong endophilic behavior [41]. Therefore, importation of ruminants infected with exotic BTV serotypes threatens not only the importing country but any European country where at risk animals would stopover [42].

Animal exportation screening is usually solely based upon the importer’s requirements. At the time of sampling, the requirements to import live ruminants into the UAE were brucellosis and Foot-and-Mouth Disease testing. The reason was mainly that most livestock would arrive by road from neighboring countries. This is of major concern, especially in cases where animals might transit in a third-party country. These results stress the need for pre-import risk assessment, precaution, and the implementation of biosecurity measures when considering the translocation of wild ruminant species susceptible to BTV and EHDV.

Therefore, we would suggest avoiding stopovers of live ruminant shipments in countries where competent orbiviruses vectors were reported or at minimum vector control in resting areas inside airports.

In addition, we recommend *Culicoides* biological surveys to be carried out in the different natural habitats and BTV and EHDV virus surveys in all susceptible species within the UAE. 

## 4. Materials and Methods

### 4.1. Sample Origin

**Figure 1:**
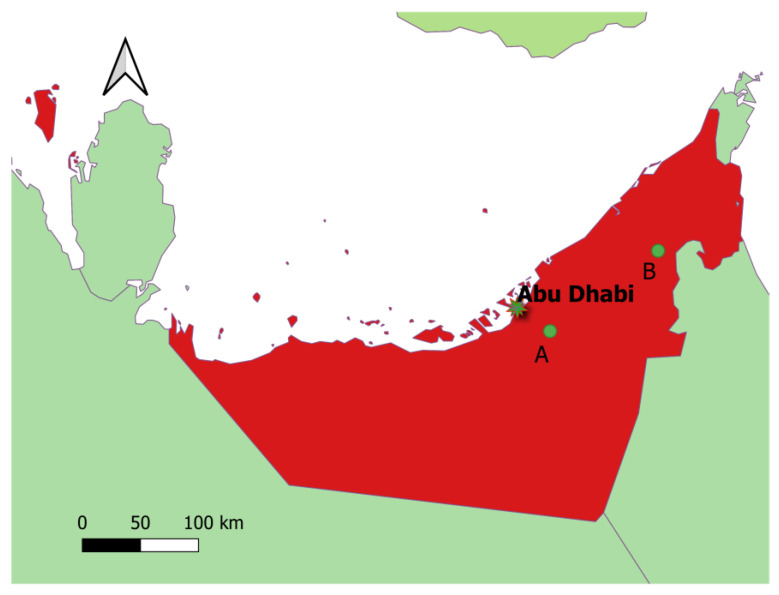
Indigenous animals sampling locations. A total of 176 out of 4000 SHOs were sampled in January and May at site A (high animal density, 5.4 km²). Nineteen out of about fifty SHOs were sampled in January 2015 at site B (semi-wild desert area, 225 km²).

One hundred and ninety-five SHOs from two locations in the UAE (Figure 1) were sampled (“indigenous animals”). In addition, 16 AOs and 13 SHOs imported from the USA (“imported animals”) were blood sampled once they arrived in the UAE.

#### 4.1.1. Indigenous Animals

For herd management and disease screening purposes, 176 SHOs were bled in January and May 2013 amongst approximately 4000 other SHOs in a high-density wildlife collection housing over 13,000 wild ungulates, located 45 km inland (24.22295, 54.76194, site A) and east of Abu Dhabi. The overall setup is approximately 5.4 km² and was already described in [43]. 

Another 19 SHOs were bled out of approximately 50 individuals in a remote and desert semi-wild location, 225 km², in Dubai emirate (24.89347, 55.66370, site B) in January 2015. Locations were about 120 km apart.

#### 4.1.2. Imported Animals

To improve the genetic diversity of the local AO and SHO populations for conservation purposes, 39 adult AOs and 42 SHOs coming from different wildlife ranches and zoological parks in the USA were gathered by an animal broker located in Texas where they underwent brucellosis and bovine tuberculosis testing required by the importer. They were flown in individual crates and transited through the European Union without being subjected to any European health requirements prior to their transit. The AOs landed on 13 October 2013 and the SHOs on 15 April 2015. They were then quarantined in a wildlife facility remotely located in the desert (24.06453, 55.04956). Sixteen AOs and thirteen SHOs were physically restrained with a chute system 15 and 5 days post-arrival, respectively. At that time, they underwent clinical examination and were blood-sampled. None of the sampled animals displayed clinical signs evocative of an *orbivirus* infection.

### 4.2. Dry Blood Spots (DBS) for Virological and Serological Testing

Blood was drawn from the jugular vein of the animals and placed into 9 mL ethylenediamine tetraacetic acid (EDTA)-coated tubes. Blood samples were then stored at −20 °C and processed within 6 months. Blood was allowed to thaw at 6 °C overnight and then placed in a dry bath set at 56 °C for 30 min to deactivate any potential Foot-and-Mouth disease virus. With a pipettor, 80 µL of blood were dispensed on Whatman protein saver cards and left out to dry at room temperature (RT) according to an adapted protocol described elsewhere [44,45]. Dry blood spots were subsequently punched out in paper discs with a 6 mm diameter punch in the middle of the deposit and diluted in 250 µL PBS and Tween 20 0.05%. Samples were then gently vortexed and left overnight at 4 °C. The obtained eluates were reported to be equivalent to a 1/25 dilution of the corresponding serum [46]. Samples were slightly stirred before using the required amount of supernatant.

### 4.3. Serology

#### 4.3.1. Epizootic Hemorrhagic Disease Virus Competitive Enzyme-Linked Immunosorbent Assay

Oryx dried blood paper discs were tested to detect antibodies against EHDV VP7 protein by competitive ELISA (LSIVet Ruminant EHDV Serum ELISA Kit, LSI, Lissieu, France) following manufacturer’s recommendations [47]. Percentage of inhibition (% inh) of each sample was interpreted as follows: % inh < 55 = negative, 55 < % inh < 60 = doubtful and % inh > 60 = positive. 

#### 4.3.2. Bluetongue Virus Indirect Enzyme-Linked Immunosorbent Assay 

Antibodies against BTV VP7 protein were tested by indirect ELISA (iELISA, ID Screen® Bluetongue Milk Indirect, ID Vet, Grabels, France) according to the manufacturer’s instructions with minor modifications. Briefly, 50 mL of supernatant and 50 mL ‘wash solution’ were added to the wells of a BTV-VP7-coated microtiter plate. After incubation for 45 min at RT, plates were washed and incubated with 100 mL anti-ruminant peroxidase conjugate for 30 min at RT. After washing, wells were incubated for 15 min at RT with 100 mL TMB substrate. Color development was stopped by the addition of 100 mL 0.5 M H_2_SO_4_. S/P% (OD_sample_/OD_positive control_ × 100%) was calculated using optical density values measured at 450 nm. S/P% ≥ 50% was considered as positive [27]. In addition to positive and negative controls from the kit, highly positive BTV-8 cattle serum and negative cattle serum from previous experiments [48] were included on each plate.

### 4.4. RT-qPCR pan-BTV (S5)

Pan-BTV RT-qPCR was performed on all imported animals (AO n = 16, SHO n = 13), whereas only seropositive samples from animals already in the UAE were tested. The detection of the BTV genome in eluted oryx samples was carried out using a pan-BTV RT-qPCR consisting of a triplex RT-qPCR (RT-qPCR) targeting segment 5, internal control (IC), and external control (EC), as described by [49]. Prior to being used on oryx samples, the RT-qPCR protocol was validated on cattle dry blood spots of known infectious status from previous experiments [50]. The RT-qPCR was performed on a LightCycler- 480 (Roche Diagnostics, Mannheim, Germany). For this assay, crossing-point values (Cp values) < 40.0 were classified as positive, Cp values >40.0 and <45.0 were classified as doubtful, and Cp values > 45.0 were considered as negative (Neg) [49]. 

### 4.5. Serotype-Specific RT-qPCR

As BTV-2 was reported in all three areas of interest (Europe, southwestern USA, and the Middle East), we focused serotype-specific detection on that particular serotype. In-house serotype-specific real-time RT-qPCRs for BTV-2 targeting segment 2 of the viral genome were carried out (cycling profile and mix set-up) similarly to the BTV serotype real-time RT-qPCRs described by Vandenbussche et al., 2009 with the extra addition of 1U Taq Platinum (Invitrogen, Merelbeke, Belgium) to the enzyme mix [51]. The following primers/probe were used, with the final concentration between brackets and LNA nucleotide with “+”: BTV-2 (forward ATGATGTTTCCAAGATTCCTGAGATG (1 µM); Reverse CATTTCGTGTTGGCATATATTTGAGTG (0.75 µM); Probe 6’FAM-CT+CA+TC+TT+TGATATCG+TAAGC-BHQ1 (0.25 µM)). 

### 4.6. Cloning/Plasmid/Preparation Sequencing

Each BTV sequence amplified by serotype-specific RT-qPCR was prepared for further sequencing. A total of 4 µL of purified serotype-specific real-time RT-qPCR fragment was ligated into the pCR2.1-Topo vector using TOPO TA Cloning (Invitrogen, Merelbeke, Belgium). Following blue/white screening on X-gal containing kanamycin (50 µg/mL) LB plates, plasmids were purified using QIAprep Spin Miniprep Kit (Qiagen, Venlo, Netherlands) according to the manufacturer’s instructions. Insert verification was carried out by Eco RI digestion and gel electrophoresis.

The purified plasmids were sequenced using the BigDye Terminator v3.1 cycle sequencing kit (Applied Biosystems, Foster City, CA, USA). The sequence reactions were purified by precipitation with 80% ethanol and centrifugation at 12,000× *g* for 15 min at 4 °C. After washing with 70% ethanol, the pellet was air-dried and dissolved subsequently in a 25 µL template suppression reagent (Applied Biosystems Foster City, CA, USA). Next, the purified product was denatured by incubation for 2 min at 94 °C and was subsequently analyzed on the ABI PRISM 310 Genetic Analyzer (Applied Biosystems, Foster City, CA, USA). The obtained sequences were identified and compared with publicly available sequences and with the obtained reference sequence using the “blast” engine at “http://www.ncbi.nlm.nih.gov/BLAST/ (accessed on 8 February 2017)” [52].

### 4.7. Statistical Analysis

The seroprevalences and their 95% confidence intervals (CI) were calculated using the binomial "exact" method in the online calculator Epitools [53].

## Figures and Tables

**Table 1:** Indirect ELISA (iELISA), competitive ELISA (cELISA), and RT-qPCR results according to the species and origin of the tested animals.

		Positive Animals/Tested Animals (% of Positive Animals; 95% CI)				
Origin of the Animals	Species	Nb of Sampled Animals/Total nb of Animals	BTV iELISA	RT-qPCR pan-BTV	RT-qPCR BTV-2	EHDV cELISA
Indigenous, site A	Scimitar Horned Oryx	176/4000	3/176 (1.7%; 0.3–4.9)	0/3	0/3	0/176
Indigenous, site B	Scimitar Horned Oryx	19/50	0/19	NA	NA	0/19
Imported (Texas)	Scimitar Horned Oryx	13/42	8/13 (61.5%; 31.6–86.1)	0/13	0/13	0/13
Imported (Texas)	Arabian Oryx	16/39	8/16 (50%; 24.7–75.4)	6/16 (38%; 18–61)	6/16	0/16

Only seropositive samples from indigenous animals were tested by RT-qPCR.

## Data Availability

Not applicable.
